# Mobile Phone–Based Use of the Photoplethysmography Technique to Detect Atrial Fibrillation in Primary Care: Diagnostic Accuracy Study of the FibriCheck App

**DOI:** 10.2196/12284

**Published:** 2019-03-27

**Authors:** Tine Proesmans, Christophe Mortelmans, Ruth Van Haelst, Frederik Verbrugge, Pieter Vandervoort, Bert Vaes

**Affiliations:** 1 Department of Cardiology Ziekenhuis Oost-Limburg Genk Belgium; 2 Department of Public Health and Primary Care University of Leuven Leuven Belgium

**Keywords:** atrial fibrillation, electrocardiography, photoplethysmography, mobile phone, algorithm

## Abstract

**Background:**

Mobile phone apps using photoplethysmography (PPG) technology through their built-in camera are becoming an attractive alternative for atrial fibrillation (AF) screening because of their low cost, convenience, and broad accessibility. However, some important questions concerning their diagnostic accuracy remain to be answered.

**Objective:**

This study tested the diagnostic accuracy of the FibriCheck AF algorithm for the detection of AF on the basis of mobile phone PPG and single-lead electrocardiography (ECG) signals.

**Methods:**

A convenience sample of patients aged 65 years and above, with or without a known history of AF, was recruited from 17 primary care facilities. Patients with an active pacemaker rhythm were excluded. A PPG signal was obtained with the rear camera of an iPhone 5S. Simultaneously, a single‑lead ECG was registered using a dermal patch with a wireless connection to the same mobile phone. PPG and single-lead ECG signals were analyzed using the FibriCheck AF algorithm. At the same time, a 12‑lead ECG was obtained and interpreted offline by independent cardiologists to determine the presence of AF.

**Results:**

A total of 45.7% (102/223) subjects were having AF. PPG signal quality was sufficient for analysis in 93% and single‑lead ECG quality was sufficient in 94% of the participants. After removing insufficient quality measurements, the sensitivity and specificity were 96% (95% CI 89%-99%) and 97% (95% CI 91%-99%) for the PPG signal versus 95% (95% CI 88%-98%) and 97% (95% CI 91%-99%) for the single‑lead ECG, respectively. False-positive results were mainly because of premature ectopic beats. PPG and single‑lead ECG techniques yielded adequate signal quality in 196 subjects and a similar diagnosis in 98.0% (192/196) subjects.

**Conclusions:**

The FibriCheck AF algorithm can accurately detect AF on the basis of mobile phone PPG and single-lead ECG signals in a primary care convenience sample.

## Introduction

### Background

Atrial fibrillation (AF) is the most common cardiac arrhythmia affecting approximately 33.5 million people worldwide [1]. AF prevalence is estimated at 3% in adults aged over 20 years, increasing in the elderly and patients with comorbid conditions such as hypertension, heart failure, coronary artery disease, heart valve disease, obesity, diabetes, and chronic kidney disease [2]. Stroke remains the most fearsome complication of AF; its risk after a diagnosis of AF is increased 5-fold [1]. Although effective anticoagulation therapy reduces this risk dramatically by 60%, initial AF episodes may frequently go undetected [3]. Indeed, contemporary studies on ischemic stroke demonstrate that AF is regularly diagnosed during or immediately after an event [4]. Importantly, AF incidence is markedly influenced by the intensity of screening efforts [5]. At the time of this study, the guidelines of the European Society of Cardiology recommended opportunistic screening in people aged 65 years and above by pulse palpation and, if irregular, by a 12-lead electrocardiogram (ECG) [6].

### Objectives

Evolving technology may offer scalability to reach a general population at relatively low cost and with minimal logistic efforts, which may further lower the threshold for screening. Mobile phones may offer an interesting modality to aid AF diagnosis as their use has exponentially increased in recent years and is continuing to grow. By applying the photoplethysmography (PPG) technique through the mobile phone camera, rhythm registration from the fingertip of a subject becomes a real possibility. A software has been developed to acquire PPG measurements with the most common mobile phones and use these signals to analyze the heart rhythm. The aim of this study was to test the diagnostic accuracy of such an approach using the FibriCheck mobile phone app (Qompium) in comparison with the gold standard method of AF diagnosis, the 12‑lead ECG.

## Methods

### Study Design

This diagnostic accuracy study was performed between October 2015 and March 2016 in 17 general practitioner (GP) centers in Belgium. Participating GPs were asked to invite patients with known paroxysmal or persistent AF to participate in the study. By searching electronic medical records, patients aged 65 years and above with a diagnosis of AF were identified. This convenience sample was supplemented with subjects without a history of AF. The presence of an active pacemaker rhythm was an exclusion criterion, as this could impact the diagnostic results obtained during the subsequent measurements. With a probability of finding a false-positive result of 5% or less (alpha=.05), an estimated AF prevalence of 50% in the study population, an expected sensitivity and specificity of 95%, and a CI of 4%, a sample size of 160 subjects was calculated. The study complies with the Declaration of Helsinki and was approved by the ethical review board of the medical faculty of the Katholieke Universiteit Leuven, Belgium (Number MP 05256). All study subjects provided written informed consent before participation. For all participants, researchers (CM, RVH) registered the demographics, vital parameters, medication use, and components of the CHA_2_ DS_2_-VASc score to determine the stroke risk (ie, congestive heart failure, hypertension, age, diabetes mellitus, previous stroke, vascular disease, and sex category).

### Photoplethysmography and the FibriCheck App

**Figure 1:**
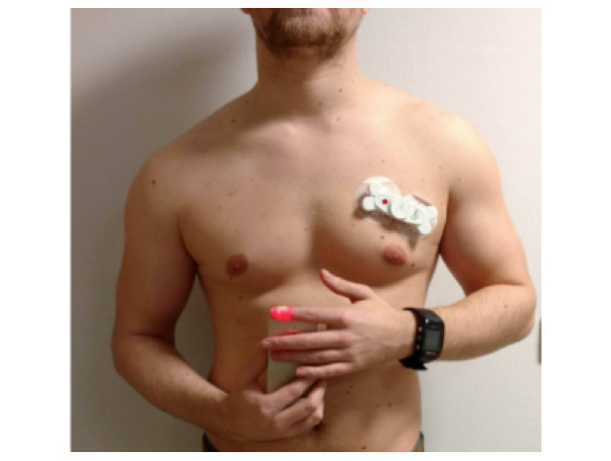
Smartphone‑based assessment of the cardiac rhythm using the FibriCheck® application. The ECG-bone, attached to a subject’s chest, for obtaining a single‑lead electrocardiogram wirelessly connected to the smartphone by the FibriCheck® application.

In every subject, a mobile phone‑based assessment of the cardiac rhythm using the Conformité Européenne–approved FibriCheck app was performed by a single researcher (CM or RVH) who was not blinded for the medical history of the patient. For this purpose, a PPG signal was acquired with the rear camera of an iPhone 5S (Apple Inc). PPG is a technique whereby a volumetric measurement is optically obtained. A classic application of the PPG technique is the pulse oximeter, which illuminates the skin and measures changes in light intensity with blood volume pulse variation in the local arterioles and uses this information to determine arterial oxygen saturation and pulse frequency. The same principle can be applied by using the camera of a mobile phone and measuring the amount of reflected light. In this way, each heartbeat is recorded, and the rhythm can be determined on the basis of the intervals between heartbeats (ie, RR-intervals). The FibriCheck app provides software to obtain and analyze such measurements with most common mobile phones. To obtain a high‑quality PPG signal, subjects were asked to adopt a sitting position with both arms resting on a table, holding the iPhone 5S in a vertical position with their right hand. Subsequently, they were asked to cover the flashlight and the rear camera horizontally with their left index finger (Figure 1). The measurement time to acquire the PPG signal with the FibriCheck app is 1 min, visualized by a countdown clock on the mobile phone screen. To minimalize motion artifacts, subjects were instructed not to speak or move during the registration process. Subjects were asked to independently perform 3 consecutive measurements. To avoid evoking a reaction following the result of a measurement, researchers and participants were blinded for the PPG signal during the measurements and the automated interpretations after the measurements. The researchers performing the measurements scored every study subject on a scale from 1 to 4 according to their experience with and handling of the mobile phone (1, optimal handling; 2, subject has good knowledge of the mobile phone and only requires minor input or corrections on handling; 3, subject has some knowledge of the mobile phone but needs substantial corrections on handling; 4, subject has never held a mobile phone before or has many issues in holding and handling it correctly).

### Single-Lead Electrocardiogram Using the Electrocardiogram-Bone

Simultaneously with the PPG measurement, a synchronized single-lead ECG was obtained using the ECG-bone (Interuniversity Micro-Electronics Center, IMEC) [7]. This module was attached with a patch on the left side of the subject’s chest above ribs 2 and 3 (Figure 1) and was wirelessly connected to the iPhone 5S with the help of the FibriCheck app. This procedure was performed by the same researcher who helped with the operation of the FibriCheck app.

### Data Processing

**Figure 2:**
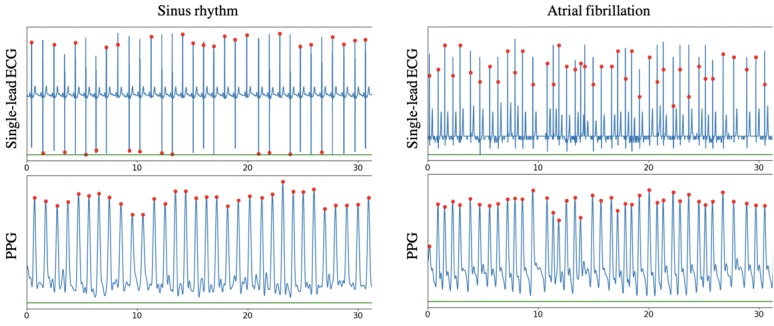
A snapshot of a synchronized photoplethysmography signal and single-lead electrocardiogram in patient with sinus rhythm (left) and atrial fibrillation (right). The red dots indicate a detected heart beat. ECG: electrocardiogram; PPG: photoplethysmography.

After simultaneous collection of both the PPG and single-lead ECG signal, data were transferred to a secured Web-based data platform for analysis. First, raw signals were analyzed by a recurrent neural network algorithm to classify them on the basis of quality metrics. The PPG signal quality judgement was based on the capacity to detect and differentiate heartbeats. If heartbeat detection was compromised with noise or if heartbeats were absent, these measurements were filtered out as insufficient quality. QRS complexes in the single-lead ECG signals were detected using the Pan-Tompkins algorithm based on slope, amplitude, and width analysis of the waveform [8]. The reliable measurements were evaluated by the FibriCheck AF algorithm on the basis of RR-interval variability analysis (Figure 2).

### Twelve-Lead Electrocardiogram

The same researcher obtained a 12-lead ECG (gold standard). The ECGs were taken using digital machines CardiMax FCP-7101 (Fukuda Denshi), CP 50 (Welch Allyn), Universal ECG (QRS Diagnostic), and ECG-1150 (Nihon Kohden Corporation) and the data were immediately printed. All 12-lead ECGs were analyzed offline on the basis of the Minnesota Code Classification System for Electrocardiographic Findings (code 8-3-1) by 2 experienced, independent cardiologists blinded to all other data. In case of a disagreement, a third cardiologist was consulted to interpret the rhythm.

### Statistical Analysis

Continuous variables are expressed as the means (SDs) if normally distributed or otherwise by medians (interquartile ranges, IQR). Categorical variables are expressed as percentages. A Mann-Whitney *U* test was used to compare mobile phone handling between patients with AF versus without AF. The levels of diagnostic accuracy of the PPG and single‑lead ECG signals analyzed by the FibriCheck AF algorithm were tested against the gold standard using 2×2 tables (MedCalc Software). Data analysis was performed both on measurement level, including the results of all 3 measurements, and on participant level, using a majority rule to determine the overall result. For both approaches, data analysis was performed (1) after exclusion of insufficient quality measurements, (2) with insufficient quality measurements categorized as *sinus rhythm*, and (3) with insufficient quality measurements categorized as *possible AF*. If 2 insufficient quality measurements were present, the majority rule did not uphold, and a decision was made on the basis of severity. The positive and negative predictive values (PPV and NPV) were also estimated on the basis of an expected AF prevalence of 6% in the population aged 65 years and above [2]. Finally, the results of PPG versus single-lead ECG were compared case by case for inconsistencies, with beat‑to‑beat analysis of the raw data to reveal the underlying reasons for any differences.

## Results

### Study Population

**Figure 3:**
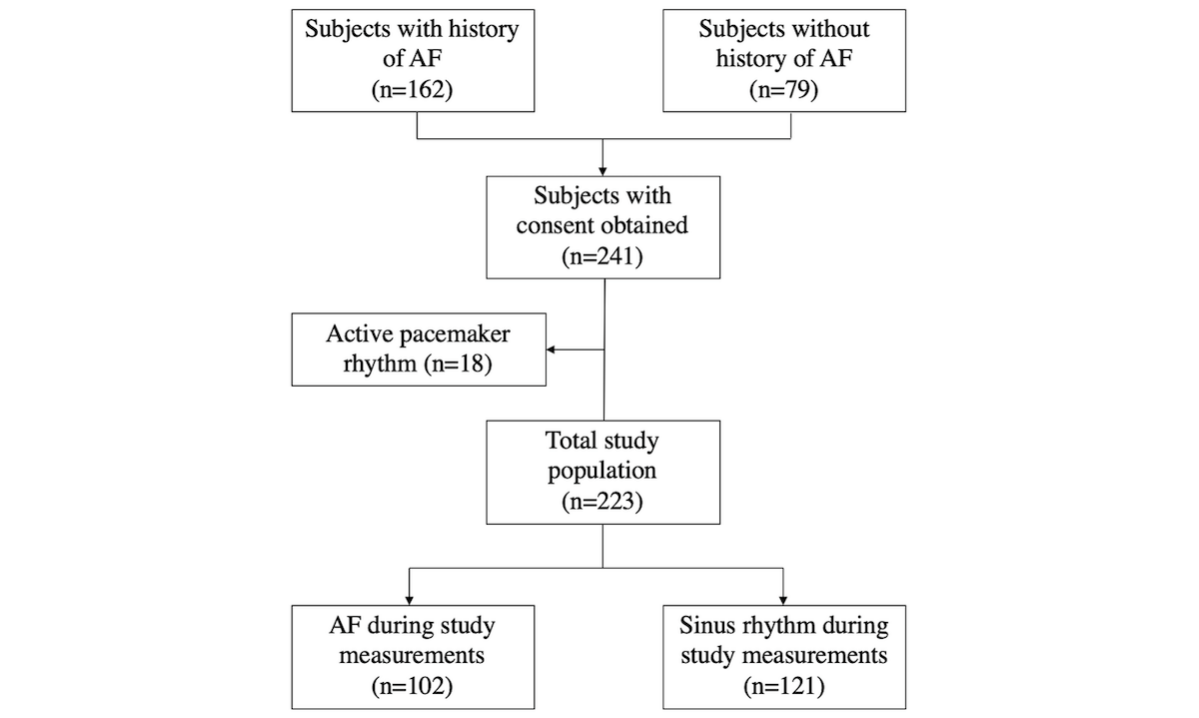
Study flowchart. AF: atrial fibrillation.

A total of 241 patients participated in the study. The study flowchart is presented in Figure 3. In total, 18 pacemaker patients had to be excluded because of active pacing during the measurements. Therefore, the final study population comprised 223 subjects. Their characteristics are presented in Table 1. Overall, the mean age was 77 (SD 8) years (range: 59 to 95 years), with 46.6% (104/223) males. AF was present in 45.7% (102/223) patients. Patients with AF had a mean CHA_2_ DS_2_‑VASc score of 5 (SD 2). Mobile phone handling was significantly different between patients with AF (median=4, IQR 3-4) versus without AF (median=3, IQR 2-4; *P*=.001).

### Photoplethysmography Measurements

#### On Participant Level

PPG measurements were recorded for a total of 223 participants. After exclusion of measurements of insufficient quality, 7% (16/223), a PPG signal suitable for analysis was obtained for 92.8% (207/223) subjects. Positive results were found in 91 subjects and negative results were found in 116 subjects. PPG results matched the diagnosis made by cardiologists on the basis of the 12-lead ECG in 96.1% (199/207) subjects, resulting in an overall sensitivity of 95.6% (95% CI 89.1%-98.8%) and a specificity of 96.6% (95% CI 91.4%-99.1%; Table 2). From the 8 inconsistent results, 4 were false-positive and 4 false-negative. False-positive results were caused by atrial premature beats (n=4). False-negative results were caused by peak wave undersensing (n=1) and misinterpretation of an atrial flutter as sinus rhythm (n=3). On the basis of an expected prevalence of 6% in the population aged 65 years and above, a PPV of 63% (95% CI 61.3%-64.8%) and an NPV of 99.7% (95% CI 99.6%-99.8%) were estimated.

**Table 1:** Characteristics of the study population (N=223).

Characteristics		Healthy patients (n=79)	AF^a^ patients with sinus rhythm (n=42)	AF patients with AF (n=102)	Total population (N=223)
Age (years), mean (SD)		75 (8)	78 (8)	79 (8)	77 (8)
Male, n (%)		32 (41)	21 (50)	51 (49.0)	104 (46.6)
Resting heart rate, bpm^b^, mean (SD)		71 (14)	70 (18)	83 (20)	77 (19)
Systolic BP^c^, mm Hg, mean (SD)		130 (16)	129 (14)	129 (17)	129 (16)
Diastolic BP, mm Hg, mean (SD)		73 (8)	74 (7)	74(11)	74 (9)
**Risk factors**					
	CHA_2_ DS_2_-VASc^d^-score, median (IQR^e^)	3 (2-4)	4 (3-5)	5 (3-6)	4 (3-6)
	Congestive heart failure, n (%)	12 (15)	10 (24)	42 (41.2)	64 (28.7)
	Diabetes, n (%)	9 (11)	9 (21)	27 (26.5)	45 (20.2)
	Stroke or transient ischemic attack, n (%)	9 (11)	9 (21)	32 (31.4)	50 (22.4)
	Atherosclerotic disease, n (%)	19 (24)	22 (52)	48 (47.1)	89 (40.0)
**Medication use**					
	Anticoagulation, n (%)	2 (3)	30 (71)	92 (90.2)	124 (55.6)
	ACE^f^ inhibitor, n (%)	11 (14)	13 (31)	32 (31.4)	56 (25.1)
	Angiotensin receptor blocker, n (%)	11 (14)	10 (24)	21 (20.6)	42 (18.8)
	Beta blocker, n (%)	33 (42)	22 (52)	71 (69.6)	126 (56.5)
	Diuretics, n (%)	16 (20)	11 (26)	53 (52.0)	80 (35.9)
**Mobile phone handling**					
	Mobile phone ownership, n (%)	19 (24)	6 (14)	11 (10.8)	36 (16.1)
	Mobile phone handling, median (IQR)	3 (2-4)	4 (2-4)	4 (3-4)	4 (2-4)

^a^AF: atrial fibrillation.

^b^bpm: beats per minute.

^c^BP: blood pressure.

^d^CHA_2_ DS_2_-VASc: congestive heart failure, hypertension, age, diabetes mellitus, previous stroke, vascular disease, and sex category.

^e^IQR: interquartile range.

^f^ACE: angiotensin‑converting enzyme.

**Table 2:** Diagnostic accuracy of photoplethysmography and single-lead electrocardiography signal analysis on participant level, based on a majority rule, compared with the reference gold standard 12-lead electrocardiography.

Diagnostic metrics	Insufficient quality excluded		Insufficient quality categorized as *sinus rhythm*^a^		Insufficient quality categorized as *possible AF*^b^	
Diagnostic test	PPG^c^ (n=207)	ECG^d^ (n=210)	PPG (n=223)	ECG (n=223)	PPG (n=223)	ECG (n=223)
Prevalence, n (%)	91 (44.0)	95 (45.2)	100 (44.8)	100 (44.8)	100 (44.8)	100 (44.8)
Sensitivity (%)	95.6	94.7	87	90	96	95
Specificity (%)	96.6	96.6	96.8	96.8	91.1	91.1
PPV^e^ (%)	95.6	95.7	95.6	95.7	89.7	89.6
NPV^f^ (%)	96.6	95.7	90.2	92.3	96.6	95.7
Accuracy (%)	96.1	95.7	92.4	93.7	93.3	92.8

^a^The rhythm categories *sinus rhythm* and *possible AF* were made by separating the measurements indicative for sinus rhythm and AF, and by adding to them insufficient quality measurements as stated in the column headings.

^b^AF: atrial fibrillation.

^c^PPG: photoplethysmography.

^d^ECG: electrocardiogram.

^e^PPV: positive predictive value.

^f^NPV: negative predictive value.

Using the same approach but classifying insufficient quality measurements as *sinus rhythm*, a sensitivity of 87% (95% CI 78.80%-92.89%) and a specificity of 96.75% (95% CI 91.88%-99.11%) were obtained (Table 2). In this scenario, PPG results matched the cardiologists’ interpretation of the 12-lead ECG in 92.4% (206/223) subjects. The amount of false-negatives in this scenario increased to 13. Classifying insufficient quality measurements as *possible AF* yielded a sensitivity of 96% (95% CI 90.07%-98.90%) and a specificity of 91.06% (95% CI 84.56%-95.45%; Table 2). Here, PPG results matched the diagnosis of 12-lead ECG in 93.2% (208/223) subjects and the number of false-positives increased to 11.

#### On Measurement Level

A total of 657 PPG measurements were recorded, 16.7% (110/657) were labelled as *insufficient quality* by the algorithm quality filter. Analyzing solely high-quality PPG measurements resulted in a sensitivity of 95.28% (95% CI 91.71%-97.62%) and a specificity of 96.18% (95% CI 93.42%-98.01%; Table 3). For 95.8% (524/547) PPG measurements, the diagnosis matched the diagnosis on the basis of the 12-lead ECG. The 23 inconsistent results were caused by 12 false-positives and 11 false-negatives. When categorizing insufficient quality as *sinus rhythm*, the sensitivity dropped to 76.03% (95% CI 70.71%-80.81%) with a specificity of 96.71% (95% CI 94.33%-98.29%; Table 3). This resulted in an agreement between PPG and 12-lead ECG for 87.5% (575/657) measurements and an increase of false-negatives to 70. Interpreting insufficient quality as *possible AF* resulted in a sensitivity and specificity of 96.23% (95% CI 93.36%-98.10%) and 82.74% (95% CI 78.46%-86.47%), respectively (Table 3). Overall, 88.7% (583/657) PPG measurements had the same diagnosis compared with 12-lead ECG. Here 11 measurements were false-negative, and 63 measurements were false-positive.

#### Insufficient Quality

The Chi-square test was used to identify causes or correlations between comorbidities and insufficient PPG measurements (Table 4).

In addition, on the basis of the Chi-square test, there is no association between mobile phone handling and insufficient quality (*P*=.43).

### Single-Lead Electrocardiogram by the Electrocardiogram-Bone

#### On Participant Level

Single-lead ECG recordings were collected from a total of 223 participants. After eliminating insufficient quality measurements, a single-lead ECG signal suitable for analysis was obtained for 94.2% (210/223) subjects. Positive results were found in 90 subjects and negative results were found in 111 subjects. Single-lead ECG results matched the diagnosis made by cardiologists on the basis of the 12-lead ECG in 95.7% (201/210) cases, yielding a sensitivity and specificity of 94.74% (95% CI 88.14%-98.27%) and 96.55% (95% CI 91.33%-99.04%), respectively (Table 2). Among the 9 inconsistent results, 4 were false-positive and 5 were false-negative. False-positive results were caused by atrial (n=3) and ventricular (n=1) premature beats. False-negative results were caused by misinterpretation of an atrial flutter as sinus rhythm (n=5).

Including the insufficient quality measurements as *sinus rhythm* resulted in a sensitivity of 90% (95% CI 82.38%-95.10%) and a specificity of 96.75% (95% CI 91.88%-99.11%), whereas including these measurements as *possible AF* resulted in a sensitivity of 95% (95% CI 88.72%-98.36%) and a specificity of 91.06% (95% CI 83.58%-94.86%; Table 2). In the first scenario, the amount of false-negatives increased to 10. In the latter, the amount of false-positives increased to 12.

**Table 3:** Diagnostic accuracy of photoplethysmography and single-lead electrocardiography signal analysis on measurement level compared with reference gold standard 12-lead electrocardiography.

Diagnostic metrics	Insufficient quality excluded		Insufficient quality categorized as *sinus rhythm*^a^		Insufficient quality categorized as *possible AF*^b^	
Diagnostic test	PPG^c^ (n=547)	ECG^d^ (n=612)	PPG (n=657)	ECG (n=657)	PPG (n=657)	ECG (n=584)
Prevalence n (%)	233 (42.6)	274 (44.8)	292 (44.4)	291 (44.3)	292 (44.4)	291 (44.3)
Sensitivity (%)	95.30	92.00	76.00	86.60	96.20	92.40
Specificity (%)	96.20	96.50	96.70	96.70	82.70	89.10
PPV^e^ (%)	94.90	95.50	94.90	95.50	81.70	87.10
NPV^f^ (%)	96.50	93.70	83.50	90.10	96.50	93.70
Accuracy (%)	95.80	94.40	87.50	92.20	88.70	90.60

^a^The rhythm categories *sinus rhythm* and *possible AF* were made by separating the measurements indicative for sinus rhythm and AF, and by adding to them insufficient quality measurements as stated in the column headings.

^b^AF: atrial fibrillation.

^c^PPG: photoplethysmography.

^d^ECG: electrocardiogram.

^e^PPV: positive predictive value.

^f^NPV: negative predictive value.

**Table 4:** The effect of comorbidities on the signal quality of photoplethysmography measurements.

Comorbidity	*P* value on measurement level	*P* value on subject level
Diabetes	.18	.15
Heart failure	.32	.73
Gender	.02	.44
Body mass index (>25)	.02	.41
Age >75 years	.06	.58
Vascular disease	<.001	.86

#### On Measurements Level

A total of 657 single-lead ECG measurements were recorded, out of which 7% (45/657) were identified as insufficient quality. Analysis of solely high-quality measurements yielded a sensitivity and specificity of 91.97% (95% CI 88.10%-94.90%) and 96.45% (95% CI 93.88%-98.15%), respectively (Table 3). There was an agreement between the diagnosis based on single- and 12-lead ECG for 94.4% (578/612) measurements. Here 12 measurements were false-positive and 22 measurements were false-negative. Categorizing the insufficient quality measurements as *sinus rhythm* resulted in 86.60% (95% CI 82.14%-90.29%) sensitivity and 96.72% (95% CI 94.34%-98.29%) specificity (Table 3). The diagnosis based on single-lead ECG matched the diagnosis based on 12-lead ECG for 92.2% (606/657) measurements. The amount of false-negative measurements increased to 39. Interpreting insufficient quality as *possible AF* resulted in a sensitivity of 92.44% (95% CI 88.87%-95.20%) and a specificity of 89.07% (95% CI 85.42%-92.08%; Table 3). Here, there was an agreement for 90.6% (595/657) measurements. The amount of false-positives increased to 40.

### Consistency Between Photoplethysmography and the Single-Lead Electrocardiogram Signals

In 87.9% (196/223) subjects, the quality of both the PPG and single-lead ECG signals were reliable for analysis. Both signals resulted in similar diagnoses in 98.0% (192/196) subjects. On measurement level, 78.7% (516/656) PPG and single-lead ECG paired measurements had a sufficient quality of reliable analysis. This resulted in similar diagnosis in 98.1% (506/516) measurements.

## Discussion

### Principal Findings

This diagnostic accuracy study in a primary care convenience sample revealed that cardiac rhythm analysis through a mobile phone–based PPG signal with the FibriCheck AF algorithm had very good sensitivity and specificity to detect AF. False-positive results were mainly because of the presence of extrasystoles. Furthermore, the FibriCheck AF algorithm accurately diagnosed AF on the basis of a single-lead ECG, with a similar sensitivity and specificity compared with the PPG signal. Both sensitivity and specificity were affected when insufficient quality measurements were included as either *sinus rhythm* or *possible AF*, leading to a decrease in accuracy to 92.38% and 93.27%, respectively, from 96.14%. Beat-to-beat analysis showed a strong agreement between the PPG and the single-lead ECG signal.

The diagnostic accuracy of the FibriCheck AF algorithm was comparable with other screening methods and devices. A recent systematic review and meta-analysis found the greatest accuracy for blood pressure monitors and non-12-lead ECGs [9]. The modified sphygmomanometers had a pooled sensitivity of 98% and a specificity of 92%. Non-12-lead ECGs scored a sensitivity of 91% and a specificity of 95%. However, when focusing on the primary care setting, a lower specificity of 89% was obtained. Mobile phone apps also showed a good pooled accuracy, with 97% sensitivity and 95% specificity. The AliveCor, a handheld single-lead ECG device, showed a sensitivity of 94% and a specificity of 99% in cardiology clinic patients [10], but AliveCor showed a low sensitivity ranging from 55% to 79% and a specificity between 97.5% and 97.9% in hospitalized patients [11]. This was later attributed to several defects, which impaired diagnostic accuracy and necessitated a product recall in the United States during the course of the study. The commercial algorithm has been biased for enhanced specificity, whereas the version of the AF detection algorithm used in published screening studies was biased for enhanced sensitivity. The defects, together with the enhanced specificity biasing, resulted in the reported low sensitivity [12].

A mobile phone app is quick, inexpensive, and practical without the need for special infrastructure or external hardware. The patient does not require any experience or medical education and can be easily trained to use the app. Physicians can remotely review the transferred data, which enable optimal patient follow-up in a less time-consuming manner. Furthermore, the high accessibility of mobile phone apps and the increasing mobile phone usage among the elderly are important assets [13,14]. However, only 17% of our study population owned a mobile phone compared with the 27% reported in recent Austrian [12] and American [14] senior surveys. Recent Belgian and Dutch surveys reported mobile phone use in 54% of the population aged between 65 and 75 years and 29% in the population aged 75 years and above [15]. Furthermore, a relatively high difficulty in mobile phone handling was observed (Table 1). However, it is expected that, together with AF prevalence, the mobile phone usage in the senior population will continue to rise and the lack of familiarity will partially fade. Moreover, a recent study demonstrated an increasing willingness and capacity to use mobile health devices by older persons [16].

This phase 2 diagnostic study demonstrates that great opportunities lie in AF screening through PPG measurements. However, the place of the FibriCheck app in future screening or case-finding programs for AF remains to be determined. The FibriCheck app could be a good candidate for implementation as a case-finding or event-recording solution for paroxysmal AF in high-risk patients in primary care or patients with paroxysmal palpitations without a clear diagnosis. Furthermore, this mobile technology also allows follow-up of patients after resynchronization or ablation. Indeed, it has been demonstrated that intermittent measurements over a longer time period, as made possible by a mobile phone app, have a great chance of increasing the diagnostic yield.

Further research, such as validation studies and cluster randomized trials, is needed to investigate the effects of these implementation strategies and the performance in a population with a lower incidence of AF.

### Limitations

This is the first study investigating the diagnostic accuracy of the FibriCheck app in a realistic primary care population. The simultaneous measurement of PPG and single-lead ECG offered the opportunity for beat-to-beat comparisons of the 2 measurement methods to reveal the underlying reasons for inconsistencies in diagnosis using the FibriCheck AF algorithm. However, a few limitations should be noted. First, different digital 12-lead ECG devices were used as the reference standard instead of 1 standardized device. Second, there was a gap of a few minutes between the simultaneous PPG and single-lead ECG measurements and the subsequent 12-lead ECG measurement, and the subject’s heart rhythm might have changed in that short time period. Third, to calculate the PPV and the NPV in a population aged over 65 years, we assumed an AF prevalence of 6%. However, because of the heterogeneity between conducted studies, various values were found for AF prevalence in the literature [2,17]. Fourth, as the study population was a convenience sample, extrapolation of these results to the general population should be made with caution. In addition, all measurements were performed under medical supervision. Although participants and researches were blinded for all notifications and results and were thereby prevented to attempt to improve the measurement results, it remains unclear whether such apps would achieve the same accuracy in an unsupervised (real-world) situation. Another important aspect that should be considered is the accuracy of the algorithm to screen patients who may have uncontrolled high heart rates. As this study was positioned as a validation study and not as a screening study, further research to assess real-life accuracy is warranted. Finally, some false-positive results with the FibriCheck AF algorithm were caused by atrial or ventricular extrasystoles, which is a known issue in AF screening using RR-interval variability analysis. However, as confirmation with 12-lead ECG or single-lead ECG documenting P-waves is required and recommended by several guidelines [7,18,19], this limitation does not jeopardize the potential of FibriCheck as screening tool.

### Conclusions

To conclude, the FibriCheck app is an accessible standalone mobile phone app that showed promising results for AF detection in a primary care convenience sample. The FibriCheck AF algorithm showed a very good sensitivity and specificity. These findings confirm the FibriCheck app to be a possible candidate to implement in future screening, case-finding programs for AF, or monitoring programs in a home setting. However, further research is needed to determine the place of the FibriCheck app in such a strategy.

## Abbreviations

AF: atrial fibrillation
ECG: electrocardiogram
GP: general practitioner
IMEC: Interuniversity Micro-Electronics Center
IQR: interquartile range
NPV: negative predictive value
PPG: photoplethysmography
PPV: positive predictive value
RR-interval: intervals between heartbeats
